# The Performance of Children Prenatally Exposed to HIV on the A-Not-B Task in Kilifi, Kenya: A Preliminary Study

**DOI:** 10.3390/ijerph10094132

**Published:** 2013-09-04

**Authors:** Amina Abubakar, Penny Holding, Anneloes Van Baar, Charles. R. J. C. Newton, Fons. J. R. Van de Vijver, Kimberly Andrews Espy

**Affiliations:** 1 Neuroassessment, Centre for Geographic Medicine Research, Coast/KEMRI Wellcome Trust Research Programme, Box 230, Kilifi 80108, Kenya; E-Mails: penny.holding@uclmail.net (P.H.); c.newton@kemri-wellcome.org (C.R.J.C.N.); 2 Department of Cross-Cultural Psychology, Tilburg University, P.O. Box 90153, Tilburg 5000 LE, The Netherlands; 3 Child and Adolescent Studies, Utrecht University, P.O. Box 80125, Utrecht, Utrecht 3508 TC, The Netherlands; E-Mail: A.L.vanBaar@uu.nl; 4 International Centre for Behavioral Studies, P.O. Box 34307, Mombasa 80118, Kenya; 5 Department of Psychiatry, University of Oxford, Oxford OX3 7JX, UK; 6 Department of Cross-Cultural Psychology, Tilburg University, P.O. Box 90153, Tilburg 5000 LE, The Netherlands; E-Mail: Fons.vandevijver@uvt.nl; 7 Department of Psychology, University of Oregon, Eugene, OR 97403, USA; E-Mail: kaespy@uoregon.edu

**Keywords:** HIV, executive functions, A-not-B task, Kenya, children

## Abstract

The aim of the study was to investigate early executive functioning in young children from 6–35 months of age. The study involved 319 randomly selected children from the community, 17 HIV exposed but uninfected children and 31 HIV infected ARV-naive children. A variation of the A-not-B task was used. While there were no group differences in *total correct*, *perseverative errors*, nor *maximum error run*, a significant percentage of children were unable to complete the task as a consequence of the children becoming overtly distressed or refusing to continue. In a multivariate analysis we observed that the significant predictors of non-completion were HIV exposure (both infected and exposed) and being under 24 months of age. These patterns of results indicate that future work with a broader array of tasks need to look at the association of HIV and EF tasks and potential contribution of factors such as emotion regulation, persistence and motivation on performance on EF tasks.

## 1. Introduction

Children born to human immunodeficiency virus (HIV) positive mothers are at risk for poor cognitive outcomes [1]. Impairments have been observed regardless of the children’s own HIV status [2]. The mother’s status may impact upon the child’s development in a number of ways: (a) vertical transmission and subsequent HIV involvement in the central nervous system; (b) compromise the prenatal environment leading to higher incidences of low-birth weight and prematurity; and (c) compromise the postnatal environment through exposure to stressors such as suboptimal stimulation at home. Executive Functions, those behaviors that enable us to exert control over more automatic responses [3], are strongly associated with the development of the pre-frontal cortex [4]. Evidence that demonstrates an association between HIV, CNS involvement and deficits in executive functioning (EF), supports the importance of a neurophysiological pathway explaining possible functional deficits, associated with a thinning of the cerebral cortex and loss of volume in the medial frontal and premotor cortices [5,6]. Evidence from a variety of outcomes, at different stages of development and in different contexts will enable a better understanding of the pathways to impaired development. This understanding will provide an evidence base for the design of possible interventions. 

Executive Functions are defined as “the control, supervisory or self-regulatory functions that organize and direct all cognitive activities (including memory, inhibitory control and set shifting), emotional responses and overt behavior” [7]. Studies in older children have consistently indicated EF deficits in the HIV population [8,9]. However, little is known about the age of onset of EF deficits since there are no published data on EF among infants exposed to HIV known to the study team [1]. The lack of inclusion of EF measures in studies on children of less than 3 years may reflect the influence of Luria’s theory which asserts that EF becomes functional at 4 years of age [10]. However, studies of healthy infants have indicated that aspects of EF can be measured as early as 6 months of age with infants manifesting rudimentary abilities of working memory, inhibitory capacity, and goal-directed behavior [11]. Moreover, EF impairments have been detected as early as 9 months among high-risk populations, such as children with preterm birth [12] or sickle cell anemia [13], or children living in extreme poverty [14]. The importance of investigating EF lies in their fundamental role in cognitive, behavioral and socio-emotional outcomes [15]. 

General developmental delays associated with HIV infection among African children are widely reported [1,16,17]; developmental delays have also been reported among HIV-exposed, uninfected children, although these findings have been inconsistent [1,2]. The purpose of this study is to identify the impact of prenatal HIV exposure on EF in infants and toddlers. Based on the previously observed developmental consequences of maternal HIV, where both HIV exposed infected and uninfected children were observed to suffer impairments in multiple domains [2], we hypothesized that both exposed-infected and exposed-uninfected groups have EF deficits when compared to a reference population. Given the observation that HIV exposure has the potential to impact on neural pathways directly associated with impairments in executive function [6], we further hypothesized that these cognitive deficits would still be evident after controlling for developmental status as evidenced by psychomotor abilities of the infants. 

## 2. Methods

### 2.1. Study Site

The study was carried out in Kilifi district, at the Kenyan coast. The HIV prevalence rates are estimated at 9% for mothers attending prenatal care [18]. A Family Health Clinic within the Kilifi District Hospital (KDH) provides comprehensive care to families of those infected with HIV/AIDS. 

### 2.2. Participants

Children qualified for inclusion in this study met the following criteria: (a) aged 6 to 35 months;(b) parents spoke Kiswahili or one of the Mijikenda dialects as their primary language; and (c) parent gave informed consent. Mothers who were HIV positive and had eligible children were identified and approached for recruitment by the attending physician at the KDH Family Health Clinic; 52 families were approached, 3 families declined and 49 agreed to participate. Children were included in the “*HIV infected*” group if they had a positive HIV antibody test when they were older than 18 months, or a polymerase chain reaction (PCR) test if they were younger than 18 months. They were included in the “*HIV exposed but unifected*” group if they were born to HIV-positive mothers but tested negative. 17 HIV exposed but uninfected children and 31 HIV infected ARV-naive children were involved in the study. The World Health Organization’s (WHO) 1990 clinical staging and case definition of HIV in resource-constrained settings was used to index the degree of HIV progression. This definition categorizes HIV from stage 1 through stage 3, reflecting progression from primary HIV infection to advanced HIV/AIDS. The distribution of disease stage in the HIV infected group was as follows: 4 were at stage 1, 21 at stage 2, and 6 at stage 3. 

Test performance was compared against *a reference population* (*N* = 319), comprising children who were part of a larger study to develop normative data for measures of early brain insult [19]. Families with eligible children were identified through a demographic surveillance database at the study site. These families were approached in their homes for permission to take part in the study. The Kenya Medical Research Institute Scientific and Ethical Committees approved the study. Written informed consent was obtained from all families. 

### 2.3. General Procedures

This was a cross-sectional study, where every child was assessed only once. Just before the assessment children were given a snack to avoid any potential negative effects of short-term hunger on test taking. Neuropsychological testing was administered to the child in the presence of the mother. To measure early executive functioning a variant of the A-not-B task was used. Psychomotor development was assessed using the Kilifi Developmental Inventory (KDI) [19]. Data from the HIV population and from the reference population were collected concurrently between 2004 and 2006. If the child showed overt signs of distress the assessment was discontinued and the mother was instructed to soothe the child before we continued. If the child became overtly distressed, inconsolable or refused to continue with the task then the whole assessment session was discontinued. 

### 2.4. Measures and Procedures

#### 2.4.1. A-Not-B

This is a simple response-shifting task where the child is required to identify the hiding place of an item. The item is hidden in view of the child in one of two locations, the choice is re-presented to the child after a short delay, and the hiding place is changed between trials. This task places demands upon learning of a stimulus-response connection, working memory, the inhibition of the learned response, and a shift to a new response [20,21]. The task is designed to elicit the A-not-B or perseverative error, where a child continues to choose a previous successful response despite observing a new hiding place. Depending upon the parameters employed, this framework has demonstrated variations in response from infants, younger than 6 months, to children 6 years of age. 

In our variant of the A-not-B we followed procedures described by Espy and colleagues [20]. The stimulus object was a small biscuit, placed on one of two dark painted circles placed on a grey wooden testing board (43 by 20 cm). Two identical cups were used to cover the circles. The biscuit was placed on a circle in view of the child, and then both circles were covered with cups simultaneously. The board was removed from sight for a specified age-dependent delay interval (6–11 months: 3 s; 12–24 months: 5 s; 25–35 months: 10 s). During the delay the child was distracted with activities such as counting, singing, and clapping. The board was then returned and the child was invited to find the treat. The response was scored as correct when the child picked up, pointed to, or reached out for the cup with the biscuit. Children who picked up the appropriate cup were allowed to keep the biscuit. If the response was incorrect, the child was *not* allowed to displace the other cup. Instead both cups were removed and the biscuit retrieved for use in the next trial. The biscuit continued to be hidden at the same location until the child correctly retrieved the reward for two consecutive trials. The biscuit was then hidden at the alternate location, continuing the procedure through the remaining 10 trials. Dependent variables were: (i) *total correct trials* (the sum of all trials in which the child selected the correct location); (ii) *perseverative errors* (the total number of errors committed after the first set of two correctly solved trials); (iii) *maximum error run* (longest string of errors). 

The A-not-B task is a well-validated measure of EF, associated with frontal lobe functioning [21,22,23]. In a study by Espy *et al.* [24], scores on the A-not-B task were observed to load on the same latent factor as other measures of EF, thus establishing criterion validity. Moreover, it has been observed that A-not-B performance was sensitive to maturational changes. 

#### 2.4.2. KDI

The KDI assesses psychomotor development in children less than 36 months of age, through a series of standardized activities measuring locomotion, manual dexterity, eye-hand coordination and balance [19]. The total score is the sum across items and is age adjusted based on data from the larger community sample (see Table 1 for means across groups). In this population the KDI has shown excellent reliability (internal consistency, test-retest and inter-rater) [19]. Evidence for the validity of KDI includes positive associations of raw scores with age and differences between risk groups in age-adjusted scores [19,25]. 

**Table 1:** Means (and standard deviations) of the various background variables.

Variables	Reference population	HIV exposed	HIV infected	*p*
Sample size (Girls)	319 (159)	17 (6)	31 (14)	
Age in months	18.84 (8.43)	17.72 (8.77)	21.10 (8.86)	0.300
Maternal education ^a^	3.40 (3.50)	4.35 (4.07)	4.80 (3.55)	0.069
Psychomotor ^b^	0.08(0.84)	0.11 (0.60)	−0.91(1.86)	0.000
Weight-for-Age	−1.24 (1.08)	−1.28 (.79)	−2.12 (1.36)	0.000

^a^ Number of years attended school; ^b^ Age adjusted mean scores.

#### 2.4.3. Weight-for-Age

Children’s weights were taken on a digital scale. The children were weighed until consistent results were obtained across two measures to at least one decimal point. Weight-for-age scores were computed using WHO standards [24]. 

#### 2.4.4. Maternal Education

Family socioeconomic status was based on maternal self report of years of schooling. We used the total number of years that the mother reported to have attended school. 

### 2.5. Data Analysis Strategies

Data were analyzed using IBM SPSS 19. Based on previous research it was expected that older children would have fewer errors and higher total correct scores. We examined this using correlational analysis. Scores obtained in different age groups are not comparable as each group had their own delay interval (6–11 months: 3 s; 12–24 months: 5 s; 25–35 months: 10 s). Therefore, scores on the A-not-B task (*total of correct trials, perseverative errors, and maximum run of errors*) were age adjusted. This was done in regression analyses. Separate analyses were conducted for each age group. In each group, A-not-B task parameters were the dependent variable and age (measured in months) was the independent variable. Regression analyses were first conducted in the community control group. Using regression weights from the community, we computed age adjusted standardized residual scores (*i.e.*, part of the scores that cannot be accounted for by age) for all the children, both community and HIV exposed groups. The standardized residual from this analysis were saved for further analysis. ANCOVA was then used to compare the groups. Due to the substantial number of children unable to establish baseline set (defined as compliance with task demands for a minimum of seven trials); we carried out a logistic regression analysis to identify predictors of non-completion. We carried out a full logistic model containing all selected covariates. We then used a backwards elimination stepwise method to generate a reduced predictive model by eliminating covariates in order from highest to lowest as determined by Wald chi-square. 

## 3. Results

### 3.1. HIV Exposure and Background Variables

Results indicated significant main effects of group (three levels: *HIV infected group*, HIV *exposed-uninfected* group and the reference population) on weight-for-age and performance on the KDI (*F*(2, 364) = 10.97, *p* < 0.001 and *F*(2, 364) = 15.27, *p* < 0.001, respectively). A post hoc analysis indicated that the *HIV infected group* scored significantly lower than the *HIV exposed group* and the *reference population* on both variables. The levels of maternal education did not differ significantly across the three study groups: *F*(2, 364) = 1.08, *p* = 0.30, Table 1 presents the sample characteristics. 

### 3.2. A-Not-B Task

**Figure 1:**
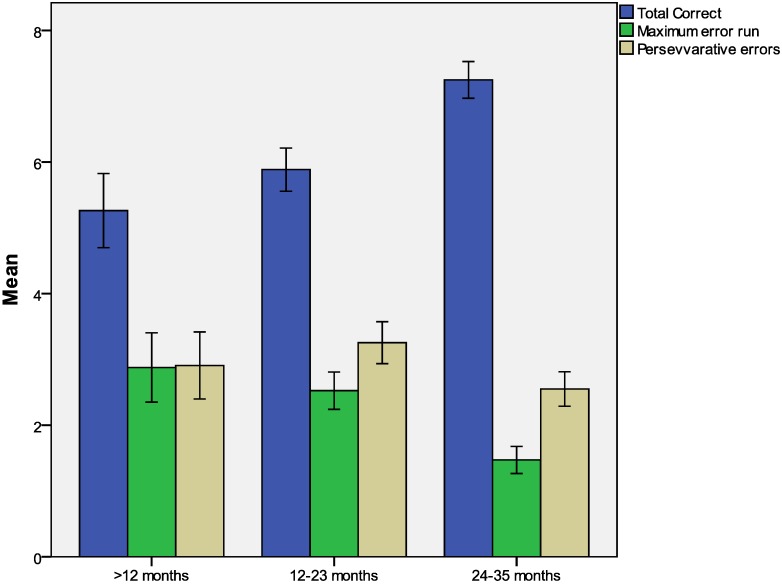
Performance of children in the A-not-B task across different age groups.

Age was significantly related to total correct (*r* = 0.467, *p* < 0.001) and maximum error run (*r* = −0.369, *p* < 0.001), but there was no significant relationship between age and perseverative errors. Figure 1 presents the performance of community children per age category in the different task parameters. This categories of age the number of children differed. For children less than 12 months *N* = 65; 12–23 months the numbers were 118 while 24–35 months there were 85 participants. What can be observed from this figure is that older children were more competent and hence had better performance compared to your children.

Table 2 presents age adjusted mean scores in the A-not-B task for each of the three groups. Analyses failed to reveal group differences in total correct, *F*(2, 292) = 0.99, *p* = 0.37, perseverative errors, *F*(2, 292) = 0.25, *p* = 0.77, or maximum error run, *F*(2, 292) = 0.34, *p* = 0.71. Entering the KDI score as a covariate yielded similar findings (total correct: *F*(3, 291) = 1.07, *p* = 0.34; perseverative errors: *F*(2, 291) = 0.22, *p* = 0.79; maximum error run: *F*(2, 291) = 0.52, *p* = 0.59). KDI scores were therefore not included in any further analysis. A significant difference was found in the number of children that completed the tasks with the HIV exposed (both infected and uninfected less likely to complete the task). χ^2^(df = 2, *N* = 367) = 11.278, *p* = 0.001; see Table 2 for the number of children unable to complete the A-not-B task per HIV group.

**Table 2:** Summary statistics for performance in the A-not-B task per group.

Variables	Reference population	HIV exposed but uninfected	HIV infected	*p ^c^*
Children not completing task *N* (%)	54 (16.9)	6 (35.3)	12 (41.9%)	0.004
Total correct M (SD) ^a,b^	−0.001 (1.74)	0.720 (2.15)	−0.1936 (1.20)	0.347
Perseverative errors M (SD) ^a,b^	−0.000 (1.70)	−0.200 (1.41)	−0.252 (1.48)	0.777
Maximum run of error M (SD) ^a,b^	0.001 (1.54)	−0.22 (1.69)	0.335 (1.01)	0.569

^a^ age adjusted mean scores; ^b^ the N in this analysis is the total sample minus those who did not complete the task *i.e.*, 265 reference population, 11 HIV exposed uninfected and 18 HIV infected. M = Means, SD = Standard Deviation. ^c^ the p-values reported here are based on the ANOVA analysis except for the first row of data which is based on a chi-square analysis.

### 3.3. Predictors of Inability to Complete A-Not-B

Logistic regression was used to examine non-completion of the A-not-B. Our results indicated that being HIV exposed (both infected and exposed uninfected), being underweight, and being younger than 24 months significantly predicted inability to complete the A-not-B task (see Table 3). Table 3 only presents regression weight of the significant predictors (the regression weights of gender, and those of children aged 12–23 months are not include since they were non-significant and excluded in the final analysis). 

**Table 3:** Results of a logistic regression analysis on the predictors of inability to complete the A-not-B task.

	B	S.E.	Odds Ratio	Sig.
Being Underweight	−0.654	0.298	0.520	0.028
Aged 24–35 months ^a^	1.270	0.378	3.560	0.001
HIV exposed uninfected ^b^	−1.083	0.554	0.339	0.050
HIV infected ^b^	−1.228	0.427	0.293	0.004

^a^ children less than 12 months of age are the reference group; ^b^ community controls are the reference population.

## 4. Discussion

The pattern of results for HIV exposed children on the A-not-B task suggests differences with unexposed children regardless of the infection status Children exposed to HIV (both infected and uninfected) were significantly less likely to complete the A-not-B task, and clinical observations suggested that underlying problems in emotional regulation and task persistence may have contributed significantly to this finding. When the HIV exposed children did not receive the reward, they became distressed and inconsolable, and showed a greater lack of persistence than children in the reference sample. However, the conventional approach to the analysis of performance on the A-not-B task, evaluating errors and total correct scores, failed to indicate differences in performance with HIV exposure [20]. Our initial sample size was already small, and was reduced still further by the loss of those children unable to complete the task. The remaining numbers may therefore have been too small to pick up subtle EF deficits. Alternatively, given the young age range EF deficits may have not yet emerged. 

Our pattern of results for the EF task differs with those from the psychomotor task where only the infected children had a poorer performance. There are two potential explanations for these results. First, HIV exposure might have a differential impact on various developmental domains, as observed in variability in sensitivity between domains of language functioning [26]. Second, our results may reflect a differential susceptibility of different domains to environmental risk factors. It is possible that children living in HIV exposed homes are more exposed to stressful family circumstances [16,27] and that heightened stress may potentially impact more on their emotional regulation and task performance [28,29]. Given the small sample size results may be a methodological or measurement artifact. More detailed investigations of EF, alongside other measures of emotional regulation and home environment are needed to explore the relative contributions of these different pathways.

This study presents the first report of early EF in the HIV population with untreated children. However, the findings are considered preliminary; due to the relatively small size of the HIV exposed subgroups and the limited assessments of EF (*i.e.*, the use of a single EF task). While our initial intention was to investigate aspects of working memory, the performance differences observed have provided preliminary data suggesting the potential impact of HIV on motivation, persistence and emotion regulation. The performances we observed may have resulted from biomedical factors (e.g., disease stage and nutritional status) or from accompanying suboptimal environmental conditions. Our study design and sample size do not allow us to evaluate this in detail. With the introduction of Highly Active Antiretroviral Therapy in Africa, the study of treatment effects upon EF development in the HIV population also warrants investigation. 

Another potential limitation of the study is the lack of testing for HIV status in the community group. Due to the unacceptability of ad hoc testing and a lack of treatment options at the time of the study, we did not test for the HIV status of children in the control group; so, this group may have included HIV-positive children. However, taking into account the prevalence rates of infection (infection rate in mothers of 9%, expected mother-child transmission rate of 25–40%, high mortality of up to 50% by the age of 2 years in the infected group) and exclusion of children from the community sample with a reported chronic disease or infection, we estimated that out of the 319 community controls at most 3–12 children were likely to be HIV positive [24]. Therefore, we expected that their impact on the test results were negligible.

## 5. Conclusions

In conclusion, this study showed that the children born to HIV positive mothers, whether infected or exposed but uninfected, may be at-risk of poor performance on challenging and novel tasks. Further studies in both HIV groups are needed to determine the nature of these deficits, their relationship to EF and to clarify the pathways involved. 
